# Skin as an immune organ and clinical applications of skin-based immunotherapy

**DOI:** 10.1186/s40413-018-0215-2

**Published:** 2018-12-07

**Authors:** J. Andrew Bird, Mario Sánchez-Borges, Ignacio J. Ansotegui, Motohiro Ebisawa, José Antonio Ortega Martell

**Affiliations:** 1 Department of Pediatrics, Division of Pediatric Allergy and Immunology, University of Texas Southwestern Medical Center, 5323 Harry Hines Boulevard, Dallas, TX 75390-9063 USA; 2 Centro Médico Docente La Trinidad and Clinica El Avila, Caracas, Venezuela; 3 Hospital Quironsalud Bizkaia, Bilbao, Spain; 4 Sagamihara National Hospital, Sagamihara, Kanagawa Japan; 5 Universidad Autónoma del Estado de Hidalgo, Pachuca, Hidalgo Mexico

**Keywords:** Allergen immunotherapy, Epicutaneous immunotherapy, Food allergy, Skin

## Abstract

**Background:**

The prevalence of food allergy is increasing, and allergen avoidance continues to be the main standard of care. There is a critical need for safe and effective forms of immunotherapy for patients with food allergy as well as other allergic diseases.

**Findings:**

The skin is a multifunctional organ with unique immunologic properties, making it a favorable administration route for allergen-specific immunotherapy. Epicutaneous immunotherapy (EPIT) takes advantage of the skin’s immune properties to modulate allergic responses and is thus one of the allergen-specific immunotherapy approaches currently being investigated for food allergy. Advances made in the understanding of how epicutaneously applied proteins interact with the immune system and in the technology for facilitating such interactions offer many opportunities for clinical application. Research has shown that allergen delivered to intact skin via EPIT is taken up in the superficial layers of the skin by Langerhans cells, avoiding passive movement of allergen through the dermis and limiting systemic circulation. EPIT brings about allergen desensitization by activating a population of regulatory T cells (Tregs) with unique properties and the potential for inducing a sustained effect as well as the possibility (seen in animal models) for protection against further sensitizations. Several clinical trials investigating the therapeutic efficacy of EPIT for treatment of peanut allergy have been completed, as well as a Phase 2 trial for treatment of milk allergy.

**Conclusions:**

Taken together, the reviewed literature supports the concept that EPIT activates the natural desensitization pathway of the skin, offering a progressive, possibly sustained response. EPIT offers a potential alternative for allergen immunotherapy that is less invasive and carries a lower risk for systemic reactions than oral immunotherapy.

## Background

A steady increase in the prevalence of allergic diseases has occurred, with approximately 30–40% of the world’s population now being affected by one or more allergic conditions [1]. Globally, 220 million to 520 million people are estimated to suffer from food allergy [1]. In the United States, findings from a 2009–2010 study of 38,480 children (infant to 18 years of age) indicated that 8% have a food allergy [2].

Although allergen avoidance (along with preparedness to manage reactions from accidental ingestion) remains the standard of care for treatment of food allergy, new forms of immunotherapy are under investigation [3]. Food allergen desensitization refers to the process of modulating the immune response to lessen the likelihood of triggering an allergic reaction in an allergic individual and/or increase the threshold of allergen that will trigger the reaction. Allergen-specific immunotherapy (IT) aims to desensitize a patient by employing exposure to the offending allergen as a treatment [3]. Allergen-specific IT has been accomplished via several different pathways: oral (OIT), sublingual (SLIT), subcutaneous (SCIT**),** and epicutaneous (EPIT) [3].

Variations of EPIT, applying an allergen onto scarified skin, have been used in the past as a treatment for allergies, and there are numerous historical reports detailing the use of skin as a conduit for immunotherapy [4]. In studies by Vallery-Radot P, et al. (1921) [5], allergen administration onto scarified skin reduced systemic allergic symptoms in subjects who were allergic to horses. In a later study, although a large number of applications were required, Pautrizel R, et al. (1957) [6] obtained excellent results in subjects with asthma after applying allergen extract onto slightly rubbed epidermis. Application of allergen drops onto heavily scarified skin in a pollinosis study by Blamoutier P, et al. (1961) [7] resulted in a reported treatment success rate of 80%. Symptom relief was obtained rapidly, allowing for co-seasonal treatment [4]. It is important to note, though, that these older EPIT approaches did not utilize the skin’s innate immune properties, but rather used scarified skin to facilitate rapid systemic absorption. Since these early results, recent advancements in the EPIT approach and technology [8, 9] have differentiated this form of allergy therapy from the historical, early reports.

The objectives of this paper are to review the evidence supporting the role of the skin as an immune organ and to discuss its involvement in potential clinical applications in allergen immunotherapy. Specifically, this article explores how the skin can be used to induce desensitization, focusing on EPIT, including its mechanism of action, and describing the latest advancements and clinical research into this form of immunotherapy.

## Findings

### Characterizing the immune properties of the skin

The goals of allergen immunotherapy are to efficiently and safely target the immune system to induce a tolerogenic response. Specifically, efficiency refers to the capacity to induce a desired tolerogenic response in a short amount of time, which is influenced by the ability to deliver an optimal dose of antigen to lymphatic organs. Safety with allergen immunotherapy is a reflection of the ability to avoid delivering allergen to highly vascularized sites during immunotherapy, thereby minimizing systemic allergic side effects [4]. With these goals in mind, an important question must be answered: what characteristics of the skin make it a favorable conduit for allergen immunotherapy?

**Fig. 1:**
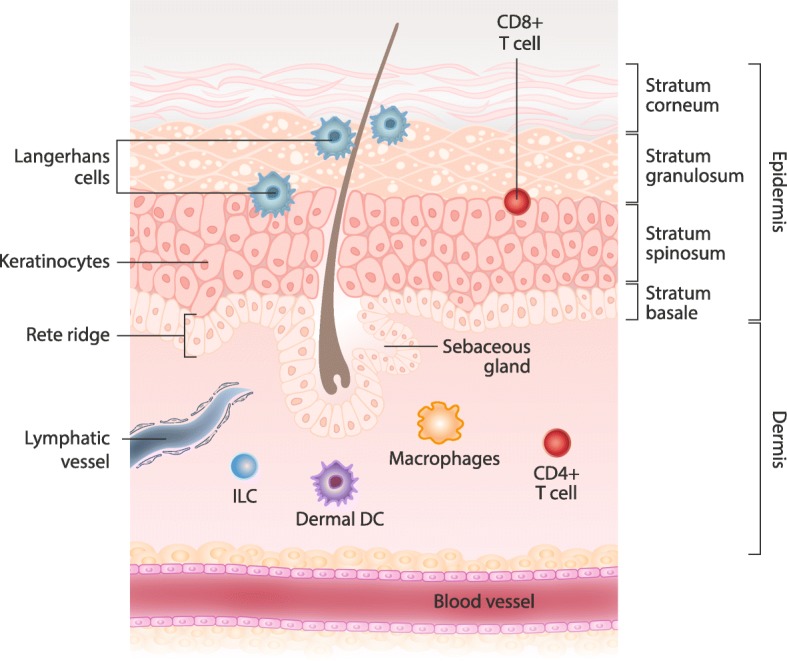
Anatomy of the Skin. CD = cluster of differentiation; DC = dendritic cell; ILC = innate lymphoid cell

The skin is a multifunctional organ under constant exposure to environmental stimuli. As such, the skin must perform numerous tasks to maintain the homeostasis needed for health. The structure of the skin therefore includes specialized cells with the ability to carry out a diverse range of functions [10]. The skin provides both a physical barrier to protect against trauma and an immunologic barrier to protect against potential pathogens. It is also a sensory-receptive area that can respond to stimuli including changes in temperature and tactile sensation, and it acts to ensure adequate hydration and protection against ultraviolet light [10]. The ability of the skin to carry out these varying functions is closely related to its structure, which consists of an outer epidermis overlying an inner dermis (Fig. 1). The epidermis is composed of 4 layers, with the stratum corneum (the outermost layer) largely responsible for providing the protective barrier between the body and the external environment [10, 11]. The dermis contains collagen fibers that provide a structural framework that hosts a dynamic immunologic environment [10, 12].

The immune functions of the skin involve “cross-talk” between the skin and the microbial environment, resulting in: 1) protective responses to trauma/injury, toxins, and infection, and 2) maintenance of self-tolerance, preventing allergic responses, and inhibiting autoimmunity [10]. The epidermis hosts two key players in the immune functions of the skin: keratinocytes and resident dendritic cells called Langerhans cells [12]. It is important to note that there are other types of dendritic cells. For example, dendritic cells found in the dermis differ from epidermal Langerhans cells, having different surface cell markers and functions [12]. Keratinocytes differentiate pathogens from harmless agents and work to mediate the immune response. They can also prompt Langerhans cells to generate an appropriate immune response [12, 13]. Langerhans cells are antigen-presenting cells in the epidermis that cluster around hair follicles, process antigens, and migrate to regional lymph nodes where they play an important role during infection and tolerance induction [12, 14, 15]. Multiple strata of keratinocytes in the epidermis make the skin a more robust barrier, with antigen passage accomplished via Langerhans cells [12, 14]. Furthermore, the epidermis is not vascularized, limiting access to the bloodstream [9]. Thus, antigen uptake in the superficial layers of the skin works to block passive conduction through the dermis and limit systemic absorption of antigens.

**Fig. 2:**
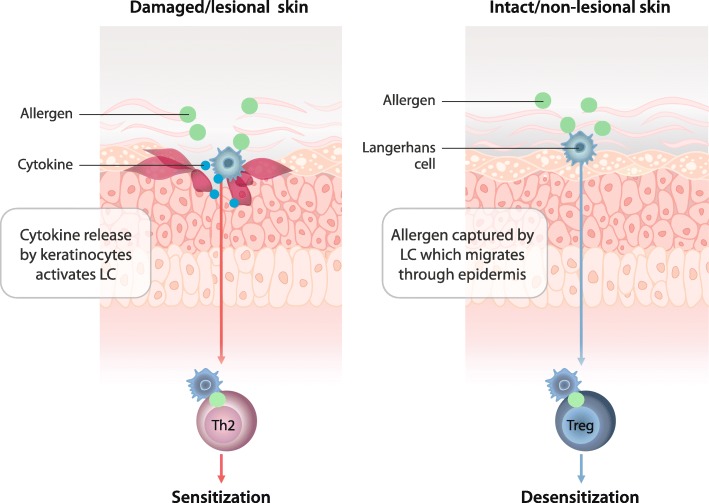
Immune Responses of the Skin. LC = Langerhans cell; Th2 = T helper cell type 2; Treg = regulatory T cell

Exposure of the skin to allergens can induce different types of immune responses that are mediated by the integrity of the skin barrier (Fig. 2). It has been previously observed that epithelial cell status instructs distinct molecular mechanisms, which promote adaptive immune responses [13, 16]. Results from a recent study also indicate that the difference in skin depth where the antigen is delivered can induce different immune responses [17]. When skin is damaged, cutaneous allergen exposure can induce sensitization (Fig. 2). More specifically, the degree of epithelial damage is key in determining the immune response, with superficial damage inducing a "noninflammatory" response transmitted by Langerhans cells and deeper epithelial damage inducing a "pro-inflammatory" response that is carried by dermal DC subsets [13]. After superficial epithelial damage, keratinocytes secrete "alarmins", such as T helper cell type 2 (Th2)-promoting cytokines, e.g., interleukin (IL)-25, IL-33, and thymic stromal lymphopoietin (TSLP), which leads to Langerhans cell activation and subsequent induction of allergen sensitization, a Th2-mediated immune response [13, 18–21]. Specifically, presentation of an allergen to naïve T cells can result in the induction of Th2 and effector cells, leading to immunoglobulin E (IgE) production and sensitization to food and environmental allergens [21, 22]. Continued exposure results in high levels of allergen-specific IgE and increased sensitization [23].

While many investigators have shown this important contribution of skin damage to the process of sensitization, others have shown that sensitization occurs even without damage in response to topical exposure [17, 24, 25]. Peanut has been shown to induce sensitization when applied to healthy intact skin through mechanisms mediated by the IL-33 receptor ST2, which result in an altered DC phenotype and a Th2- response, thereby activating the skin’s innate immunity [25]. Transdermal sensitization (to hazelnut) in intact skin has also resulted in the production of IL-4, IL-5, and IL-13, leading to IgE antibody response and clinical symptoms of anaphylaxis including intestinal damage [24]. It has been observed that 72% to 81% of peanut allergy presentations occur on first known exposure to the allergen, implying a prior occult sensitization which may include the skin [26–28]. All together, these studies support the hypothesis that skin (atopic or healthy) can be a relevant route of primary exposure to allergens (whether peanut, tree nuts, or milk) and thus, skin exposure is an important risk factor for sensitization [17, 24–26, 29, 30].

In contrast, application of allergen to intact skin has also demonstrated suppression of oral allergen sensitization and subsequent induction of desensitization (Fig. 2) [31]. Hydration of the stratum corneum enhances allergen uptake by increasing permeability while avoiding the release of pro-inflammatory cytokines [13, 32]. In intact skin, tolerogenic Langerhans cells can activate the adaptive immune system by inducing Tregs. Specifically, Langerhans cells present the antigen to T cells to induce a Treg-mediated immune response, resulting in suppression of an allergic inflammatory response and leading to desensitization [22]. Keeping in mind that the skin can be a route of exposure to allergens, epidemiological observations have also shown that low levels of environmental exposure to allergens such as peanut may be protective against sensitization [26]. Thus, application of allergen to non-lesional, intact skin may promote desensitization.

### Epicutaneous immunotherapy (EPIT) mechanism of action

**Fig. 3:**
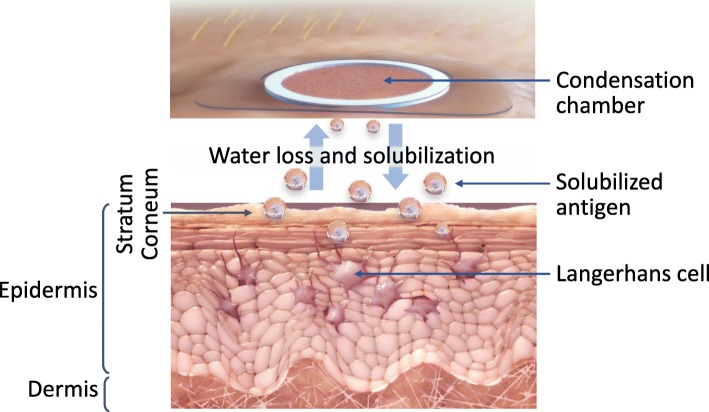
How Does EPIT Work? [38]

The latest EPIT technology utilizes the skin’s immune properties to induce desensitization [33, 34]. EPIT involves embedding the allergen onto a patch with a surrounding adhesive. DBV Technologies has developed Viaskin®, a proprietary epicutaneous electrostatic patch that promotes diffusion of allergen in the thickness of the stratum corneum and toward the immune cells of the epidermis without any skin preparation or adjuvant [8]. This patch is affixed to the skin, and because of the humidity created in the condensation chamber, the allergen diffuses into the epidermis (Fig. 3) [3]. Application of allergen to intact skin using EPIT allows for long-lasting contact of the allergen with the organism, such as with SCIT or OIT, but is less invasive, without the need for injection or swallowing of an allergen [35]. Thus, protection from vascular exposure using EPIT is an important differentiating feature in comparison to these other methods of immunotherapy [4, 35].

As observed in a sensitized mouse model, allergen delivered to intact skin via EPIT is captured in the epidermis by Langerhans cells that then migrate to lymph nodes to activate the immune system [33]. Unlike SCIT, EPIT produces no detectable systemic circulation of the allergen [36]. Furthermore, because EPIT is applied onto intact skin, as opposed to stripped skin, the allergen does not pass through activated keratinocytes or into the dermis, thus avoiding sensitization (Fig. 2) [33]. This advantage was demonstrated in a mouse model of peanut allergy in which EPIT applied to intact skin induced a tolerogenic immune profile, as exhibited by decreases in IgE and increases in immunoglobulin G (IgG) 2a production [36]. EPIT also reduced Th2 cytokine production via reduced levels of IL-4, IL-5, and IL-13 [36].

Research has shown that Tregs are at the core of the immune regulatory effect induced via EPIT. In a 2014 study, Dioszeghy and colleagues demonstrated that in a peanut-sensitized mouse model treated with EPIT, injection with anti-CD25 antibody (which depletes Tregs) increased eosinophilic infiltration. Thus, inhibition of Tregs erased the beneficial effect of EPIT, indicating a pivotal role for Tregs in inducing desensitization [37]. A more recent study by this group also supports the finding that EPIT-induced Tregs inhibit Th2 cells. EPIT-induced Tregs also express a large repertoire of homing receptors suggesting that Tregs are able to migrate to various sites of allergen exposure (ie, skin, lung, and gut), suppress local immune responses to allergen stimulation, and potentially induce global tolerance. In a peanut-sensitized mouse model, EPIT, like OIT and SLIT, significantly decreased the production of Th2 cytokines, IL-5, and IL-13, and did not result in an increase in T helper cell type 1 (Th1) cytokine expression (IFN-γ).

It is important to note, however, that the mechanism of desensitization characteristic of EPIT differs from that of OIT and SLIT. Specifically, different immunotherapies induce specific subsets of Tregs that vary in their cytokine production, surface marker expression, and mechanism of suppressing immune responses. Results from the mouse model show that EPIT and OIT increased Foxp3+ and LAP+ Tregs, while SLIT increased CD4+CD25+IL10+ Tregs. Furthermore, while all three methods induced Tregs, which contributed to the inhibition of Th2 cells, Tregs induced by EPIT required cytotoxic T-lymphocyte-associated protein 4 (CTLA-4) surface marker expression but not IL-10, OIT acted through both IL-10 and CTLA-4, and SLIT was dependent on IL-10 alone [34]. In summary, EPIT functions by activating the natural desensitization pathway of the skin.

### Review of potential applications of the skin as an immune organ and EPIT

By taking advantage of the immune properties of the skin, EPIT represents a promising form of immunotherapy and method of inducing potential food desensitization with ease of use that is less invasive and decreases the risk of systemic reactions compared with other immunotherapy approaches [3, 35, 38]. Since the historical investigations into using the skin as the route of immunotherapy administration and the preclinical studies researching the EPIT mechanism of action, several clinical development trials investigating EPIT in food allergy have been published or are currently underway.

In a pilot study for cow’s milk allergy in 18 children, EPIT using Viaskin® was found to be safe, with no reports of serious systemic adverse events. The study also demonstrated a trend toward efficacy in desensitizing children with cow’s milk allergy, warranting further investigations [39]. A Phase 1/2 trial (NCT02223182) investigating the efficacy and safety of EPIT for milk allergy (Viaskin® milk patch 150, 300 or 500 μg) in two age groups (children aged 2 to 11 and adolescents aged 12 to 17) recently reported positive preliminary Phase 2 results. A significant desensitization to milk was observed in the overall population treated with the 300 μg milk patch for 12 months, response rate = 49.0%, vs. placebo response rate = 30.2% (*P* = 0.027). For children aged 2 to 11, there was a response rate of 57.9% for the 300 μg dose vs. 32.5% for placebo (*P* = 0.042). Treatment with the milk patch was reported to be well tolerated across all doses with no treatment-related serious adverse events. Thus, milk EPIT (Viaskin® milk patch 300 μg) shows potential as the first treatment for patients suffering from IgE-mediated cow’s milk protein allergy [40].

A Phase 1 study evaluated the safety and tolerability of EPIT for peanut allergy in 100 subjects aged 6–50 years. Results showed that peanut EPIT administered on intact skin was safe and well tolerated, with high adherence [41]. Peanut EPIT was subsequently investigated in two multicenter, double-blind, randomized, placebo-controlled Phase 2 trials. The first study compared two doses of peanut EPIT (Viaskin® 100 μg and 250 μg) after 52 weeks of therapy in 74 subjects (ages 4–25 years) vs. placebo. The primary outcome, defined as passing a 5044-mg peanut protein oral double-blind, placebo-controlled food challenge or achieving a 10-fold or greater increase in successfully consumed dose from baseline to Week 52, was met. In addition, treatment was well tolerated and associated with significant immunologic and clinical responses [42]. In a Phase 2b multicenter, double-blind, placebo-controlled, dose-ranging trial with a 2-year open-label extension following 12 months of therapy, 221 subjects aged 6–55 years were randomized to placebo or Viaskin® peanut patch (50, 100 or 250 μg). The primary efficacy end point was percentage of treatment responders (eliciting dose: ≥10-times increase and/or reaching ≥1000 mg of peanut protein) in each group vs placebo patch after 12 months. Secondary end points included the percentage of responders by age strata and treatment-emergent adverse events. Subjects who received treatment with the 250-μg peanut patch demonstrated significant treatment response rates (50.0%) compared with those receiving the placebo patch (25%; difference, 25.0%; 95% CI, 7.7–42.3%; *P* = 0.01). In the open-label phase of the trial, all enrolled patients were transitioned to the 250-μg peanut patch for the remainder of the study and a progressive treatment response rate was observed at months 12 (59.7%) and 24 (64.5%) in the overall population [43].

EPIT has also been investigated for the treatment of patients with rhinoconjunctivitis using a different technology involving application on tape-stripped skin with the goal of more rapid systemic absorption [9]. In a Phase 1/2 randomized, placebo-controlled, double-blind trial of 37 subjects aged 18–65 years, grass pollen EPIT was well tolerated and resulted in significantly greater symptom improvement than seen with placebo [9]. In another Phase 1/2 double-blind, placebo-controlled dose escalation study of 132 subjects aged 18–65 years, grass pollen EPIT was safe and efficacious in a dose-dependent manner [4, 44]. Of the 3 types of skin to which EPIT can be applied – intact, tape-stripped, or scarified – allergen penetration into the dermis is blocked by intact skin, and the tape-stripping method has reduced allergen penetration into the dermis in comparison with scarification. In contrast to the immune responses triggered by allergen exposure in intact skin (Fig. 2), which feature a Treg-mediated suppression of the allergic response, tape-stripping removes the stratum corneum and application of antigen induces a systemic Th2 immune response resulting in high levels of IgE production and allergic sensitization [21]. Contrastingly, the Senti et al. grass pollen studies support that tape-stripped skin and antigen application can also be pro-tolerogenic. Tape stripping of the skin followed by EPIT results in keratinocyte secretion of pro-inflammatory cytokines (IL-1, IL-6, IL-8, TNF-α, IL-12, and IFN-γ), increased expression of MHC class II, CD86, CD40, CD54 and CD11c on Langerhans cells, enhanced Toll-like receptor 9 expression in keratinocytes, [21, 45–51] and, in these EPIT grass pollen studies, subsequently resulted in allergy-protective responses [9]. However, in EPIT with tape-stripped skin, as applied in these grass pollen studies, the mechanisms leading to allergy-protective responses and therapeutic efficacy remain unclear [13, 52].

Looking ahead, investigations are continuing into clinical applications of EPIT in intact skin. Two Phase 3 trials for peanut EPIT have been conducted, and results have been released. The recently completed Phase 3 trial evaluating the safety and efficacy of the 250-μg Viaskin® peanut patch enrolled 356 children aged 4–11 years for 12 months of blinded therapy. EPIT produced a statistically significant response with a majority of patients showing improvement in thresholds. Response rates after 12 months of treatment, based on prespecified criteria, were 35.3% in the 250-μg peanut patch group and 13.6% in the placebo patch group (difference in response rates = 21.7%; *P* < 0.001; 95% confidence interval = 12.4–29.8%). Safety and tolerability data were favorable and consistent overall with results from previous Phase 2b studies [53]. Most commonly reported treatment-emergent adverse events were mild to moderate application site reactions, and no imbalances in serious adverse events were observed. The open-label, follow-up study for this Phase 3 trial is currently underway and will evaluate the long-term efficacy and safety of 250-μg peanut patch for a total of 36 months of active treatment (NCT03013517). In another Phase 3 trial assessing the safety of Viaskin® 250-μg peanut patch in routine medical practice (without an oral food challenge as part of the study protocol, which has allowed for the entry of patients with a history of severe anaphylaxis), 393 subjects, 4–11 years of age, were enrolled. Top-line results showed a favorable safety and tolerability profile, which was comparable to data from other trials with the peanut patch, with no new or unexpected adverse events reported [54]. The open-label portion of the trial, which will monitor patients for a total of up to 36 months of active treatment, is currently underway.

Additional ongoing studies investigating EPIT for food allergy include: a Phase 3 trial evaluating the safety and efficacy of EPIT in peanut-allergic children 1–3 years of age (NCT03211247); and a Phase 2 single-site, double-blind, placebo-controlled trial to study the efficacy and safety of milk EPIT for children with milk-induced eosinophilic esophagitis (EoE) (NCT02579876).

Among the multiple forms of allergen-specific immunotherapy currently being investigated, EPIT is a novel treatment alternative being regarded as a convenient and potentially safer approach for food allergies using very low exposure doses [38, 55, 56]. While there has been great progress in the development of EPIT, OIT, and SLIT in recent years, ongoing and future studies will continue to elucidate the efficacy, safety, and treatment duration of such therapies. With the advent of various treatment options available for food allergies, the advantages of each of the therapies and their unique pathways for desensitization will be important considerations.

## Conclusions

The skin is a multifunctional organ with unique immune properties, making it a promising administration route for allergen-specific immunotherapy. EPIT, which works by activating the natural desensitization pathway of the skin, offers an alternative for allergen immunotherapy with potential ease of use and a favorable safety profile. Advancements made in the understanding of how epicutaneously applied proteins interact with the immune system and the technology for delivering such therapy offer many opportunities for clinical application.

## Abbreviations

CI: Confidence interval
CTLA-4: Cytotoxic T-lymphocyte-associated protein 4
DC: Dendritic cell
EoE: Eosinophilic esophagitis
EPIT: Epicutaneous immunotherapy
IFN: interferon
IgE: Immunoglobulin E
IgG: Immunoglobulin G
IL: Interleukin
LC: Langerhans cell
MHC: Major histocompatibility complex
OIT: Oral immunotherapy
SCIT: Subcutaneous immunotherapy
SLIT: Sublingual immunotherapy
Th1: T helper cell type 1
Th2: T helper cell type 2
TNF: Tumor necrosis factor
Treg: Regulatory T cell
TSLP: Thymic stromal lymphopoietin
